# Functional implications of hexameric assembly of RraA proteins from *Vibrio vulnificus*

**DOI:** 10.1371/journal.pone.0190064

**Published:** 2017-12-20

**Authors:** Saemee Song, Seokho Hong, Jinyang Jang, Ji-Hyun Yeom, Nohra Park, Jaejin Lee, Yeri Lim, Jun-Yeong Jeon, Hyung-Kyoon Choi, Minho Lee, Nam-Chul Ha, Kangseok Lee

**Affiliations:** 1 Department of Agricultural Biotechnology, Seoul National University, Seoul, Republic of Korea; 2 Department of Molecular Biosciences, Northwestern University, Evanston, Illinois, United States of America; 3 Department of Life Science, Chung-Ang University, Seoul, Republic of Korea; 4 School of Pharmacy, Chung-Ang University, Seoul, Republic of Korea; Centre National de la Recherche Scientifique, Aix-Marseille Université, FRANCE

## Abstract

RNase E has a pivotal role in the degradation and processing of RNAs in *Escherichia coli*, and protein inhibitors RraA and RraB control its enzymatic activity. The halophilic pathogenic bacterium *Vibrio vulnificus* also expresses orthologs of RNase E and RraA—RNase EV, RraAV1, and RraAV2 (herein renamed as VvRNase E, VvRraA1, and VvRraA2). A previous study showed that VvRraA1 actively inhibits the ribonucleolytic activity of VvRNase E by interacting with the C-terminal region of VvRNase E. However, the molecular mechanism underlying the effect of VvRraA1 on the ribonucleolytic activity of VvRNase E has not yet been elucidated. In this study, we report that the oligomer formation of VvRraA proteins affects binding efficiency to VvRNase E as well as inhibitory activity on VvRNase E action. The hexameric structure of VvRraA1 was converted to lower oligomeric forms when the Cys 9 residue was substituted with an Asp residue (VvRraA1-C9D), showing decreased inhibitory activity of VvRraA1 on VvRNase E *in vivo*. These results indicated that the intermolecular disulfide linkage contributed critically to the hexamerization of VvRraA1 for its proper function. On the contrary, the VvRraA2 that existed in a trimeric state did not bind to or inhibit VvRNase E. An *in vitro* cleavage assay further showed the reduced inhibitory effect of VvRraA-C9D on VvRNase E activity compared to wild-type VvRraA1. These findings provide insight into how VvRraA proteins can regulate VvRNase E action on its substrate RNA in *V*. *vulnificus*. In addition, based on structural and functional comparison of RraA homologs, we suggest that hexameric assembly of RraA homologs may well be required for their action on RNase E-like proteins.

## Introduction

RNase E is an essential endoribonuclease, which was initially discovered as an enzyme that is involved in the processing of ribosomal RNA in *Escherichia coli* [1]. It is now well known for its role in mRNA decay, the processing of tRNA and rRNA, and the regulation of ColE1-type plasmid replication [1–3]. The N-terminal region has the catalytic site for RNase E endonucleolytic activity and the C-terminal half (CTH) provides a platform for the interaction of multiple proteins that form a complex termed the “degradosome” together with RNase E. The association of RNase E with other enzymes in the RNA degradosome complex enables RNase E to act efficiently even when the target sites of the RNA substrates are well-structured like stem-loops [4].

The ribonucleolytic activity and intracellular concentration of RNase E are strictly regulated via several mechanisms in *E*. *coli*. The depletion, adventitious overexpression, or increased enzymatic activity of RNase E causes growth retardation of cells [5–7]. Thus, the concentration of RNase E is maintained relatively stable *in vivo* through the autoregulatory mechanism such that the enzyme cleaves the 5′-untranslated region of its own mRNA when its activity exceeds cellular needs [6, 8]. The endonucleolytic activity of RNase E is controlled by protein inhibitors RraA and RraB (regulator of ribonuclease activity A or B). They bind to separate sites in the CTH and repress the activity of RNase E. Two proteins exert distinct effects on the composition of the degradosome complex [2, 9].

**Fig 1:**
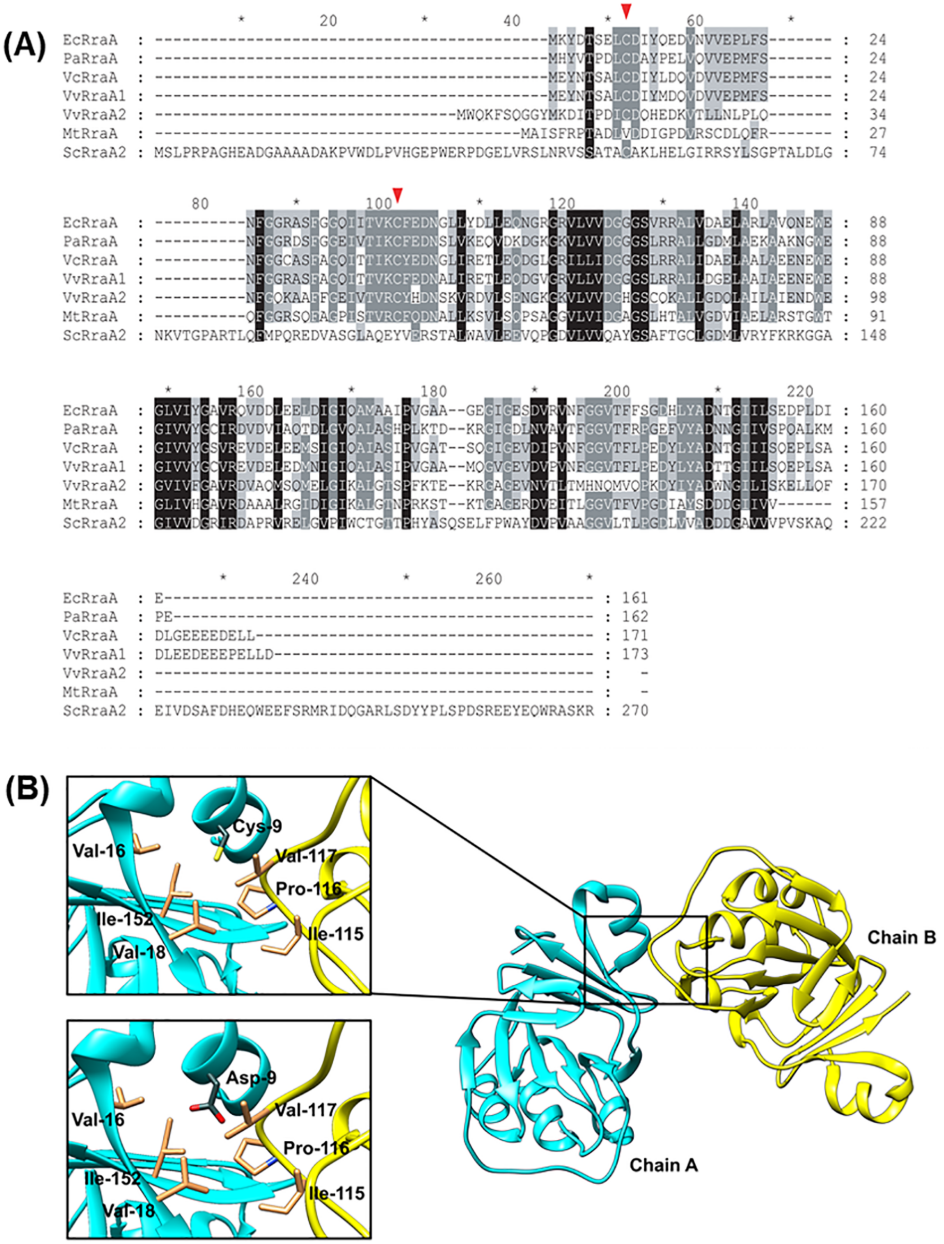
Alignment of amino acid sequences of *E*. *coli* RraA and its orthologs in Gram-negative bacteria. (A) Alignment of amino acid sequence using CLUSTAL W. VvRraA1 and VvRraA2; Amino acid sequences of RraA homologs from *E*. *coli* (EcRraA), *Mycobacterium tuberculosis* (MtRraA), *P*. *aeruginosa* (PaRraA), *Vibrio cholerae* (VcRraA), *V*. *vulnificus* (VvRraA1 and VvRraA2) are used. Arrows indicate conserved Cys9 and Cys41 residues of RraA proteins. (B) A molecular model for the C9D mutant of *E*. *coli* RraA. The model of the mutant protein was built based on the wild-type structure of *E*. *coli* RraA (PDB code: 1Q5X). The subunits are displayed in different colors (cyan and yellow). The mutated Asp9 is positioned in the hydrophobic pocket lined with residues in gold at the interface between the two neighboring subunits, which would destabilize the oligomeric forms of the protein (left lower box). The near region of Cys9 structure is shown in the left upper box.

RraA, 17.4 kDa, is an evolutionarily conserved protein found not only in bacteria but also in Archaea, proteobacteria, and plants [10]. RraA binds to the RNA-binding region in the degradosome-forming domain of RNase E in the CTH. This binding alters the composition of the RNA degradosome complex, leading to subsequent repression of the RNase E activity [2, 11, 12]. To date, there are six reported crystal structures of RraA: EcRraA (from *E*. *coli*), TtRraA (from *Thermus thermophilus*), MtRraA (from *Mycobacterium tuberculosis*), VcRraA (from *Vibrio cholerae*), PaRraA (from *Pseudomonas aeruginosa*), and ScRraA2 (from *Streptomyces coelicolor*, previously designated as RraAS2) [10, 13–17]. The primary structures of these RraA homologs share more than 29% and 44% amino acid identity and similarity, respectively with *E*. *coli* RraA (Fig 1A). The amino acid sequences were similar in the core conserved regions and the crystal structures share a ring-like homotrimeric assembly. PaRraA and ScRraA2 show additional homotrimerization interactions to form the hexamer [16, 17]. Although the structure of RraA has been determined, how the oligomerization state of RraA in solution affects the function of RraA remains unclear.

The halophilic pathogenic bacterium *V*. *vulnificus* has orthologs of *E*. *coli* RNase E and two RraA-like proteins, herein renamed as VvRNase E, VvRraA1, and VvRraA2. The primary amino acid sequence of VvRNase E reveals 86.4% similarity with RNase E, and VvRraA1 and VvRraA2 have 80.1% and 59% amino acid sequence similarity with RraA, respectively [6]. Recent studies showed that VvRNase E has conserved enzymatic properties and VvRraA1efficiently inhibits the activity of both RNase E and VvRNase E [6, 18, 19].

In this study, we investigated structural properties of VvRraA1 and VvRraA2 in order to elucidate their inhibitory action on VvRNase E.

## Materials and methods

### Strains and plasmids

Plasmids and strains used in this study are listed in Table 1. The construction of an *E*. *coli* strain that contains a deletion in the *rne* gene and expresses full-length VvRraA1 (DK001) has been described previously [6]. The *E*. *coli* strains C43 (DE3) (Lucigen) and BL21 (DE3) were used for protein expression. The full-length Vv*rraA1*, Vv*rraA2*, and Vv*rne* genes from the *V*. *vulnificus* MO6-24/O genome were PCR (polymerase chain reaction)-amplified with the proper sequence for enzyme digestion and ligated to the expression vectors pET28a, pPROEX-HTA, and pGEX-KG, respectively, using restriction enzyme sites NcoI/XhoI for Vv*rraA1* and Vv*rraAV2* and EcoRI/HindIII for Vv*rne*. To introduce the mutation on Cys 9 of VvRraA1 and VvRraA2, site-directed mutagenesis was carried out by using a QuikChange site-directed mutagenesis kit (Stratagene, USA) following the manufacturer’s instruction. The plasmids were designated as pET28a-RraAV1, pET28a-RraAV1-C9D, pPROEX-RraAV2, pPROEX-RraAV2-C9D, and pGEX-RNEV. For functional genetic studies, pKAN6B plasmid [20] was used. pKAN6B-EcRraA and pKAN6B-VvRraA1 were previously constructed [19, 20]. DNA sequences encoding VvRraA1-C9D, VvRraA2, and VvRraA2-C9D within the plasmids mentioned above were PCR-amplified with sequences for C-terminal hexahistidine tag and the enzyme site for ligation using primers RraAV1(NdeI)F and RraAV1-his(XbaI)R or primers RraAV2(NdeI)F and RraAV2-his(XbaI)R. DNA fragments were digested with NdeI and XbaI restriction enzymes and ligated into the same site on pKAN6B. Primers used in this study are listed in Table 2.

**Table 1:** List of strains and plasmids used in the present work.

Strain or plasmid	Description	Source of reference
DK001	*lacZ43 relA1 spoT*1 *thi-1 rne*::*cat recA*::*Tn10* [pLAC-RNEV2]	[6]
C43 (DE3)	F- *ompT hsdSB (rB- mB-) gal dcm* (DE3)	Lucigen
pLAC-RNEV2	pSC101 *ori*, Amp^r^, *Vvrne* under placUV5	[6]
pKAN6B-VvRraA1	p15A *ori*, Km^r^, *VvrraA1-6His* under pBAD	[19]
pKAN6B-VvRraA1-C9D	p15A *ori*, Km^r^, *VvrraA1-C9D-6His* under pBAD	Present study
pKAN6B-VvRraA2	p15A *ori*, Km^r^, *VvrraA2-6His* under pBAD	Present study
pKAN6B-VvRraA2-C9D	p15A *ori*, Km^r^, *VvrraA2-C9D-6His* under pBAD	Present study
pGEX-VvRNE	pBR322 *ori*, Amp^r^, *VvRne* with N-terminal GST-tag under *tac* promoter	Present study
pET28a-VvRraA1	f1 *ori*, Km^r^, *VvrraA1*under T7 *lac* promoter	Present study
pET28a-VvRraA1-C9D	f1 *ori*, Km^r^, *VvrraA1-C9D* under T7 *lac* promoter	Present study
pPROEX-VvRraA2	Amp^r^, *VvrraA2* under *trc* promoter with N-terminal 6-His tag	Present study
pPROEX-VvRraA2-C9D	Amp^r^, *VvrraA2*-C9D under *trc* promoter with N-terminal 6-His tag	Present study

**Table 2:** List of primers used in the present study.

Primer	Sequences (5’-3’)
rpsO5’RT	GTACACTGGGATCGCTGAATT
rpsO3’RT	GGCCCCCTTTTCTGAAACTCG
RraAV1(NdeI)F	AATTCATATGGAATACAACACTTCA
RraAV1-his(XbaI)R	AATTTCTAGATCAGTGGTGGTGGTGGTG
RraAV2(NdeI)F	AATTCATATGGGTAAGGACATTACC
RraAV2-his(XbaI)R	AATTTCTAGATTAGTGATGGTGATGGTGATGGAATTGAAGCAACTCTTTGGAAAT

### RNA preparation and reverse transcription (RT)-PCR

DK001 cells harboring pKAN6B, pKAN6B-VvRraA1, pKAN6B-VvRraA1-C9D, pKAN6B- VvRraA2, or pKAN6B-VvRraA2-C9D were grown to an OD_600_ of 0.1~0.2 containing 10 μM IPTG, and then 1 mM IPTG, except for DK001 pKAN6B, and 0.2% arabinose were added. The cultures were further grown to an OD_600_ of 1.5 and harvested to obtain total RNA. Total RNA was isolated using a PureLink^®^ RNA Mini Kit (Invitrogen). Next, synthesis of *rpsO* cDNA was performed using a Prime Script first-strand cDNA synthesis kit (Takara). The synthesized cDNA was amplified using two primers and the sequences are listed in Table 2.

### Protein purification

For protein purification, pET28a-VvRraA1, pET28a-VvRraA1-C9D, pPROEX-VvRraA2, pPROEX-VvRraA2-C9D, and pGEX-VvRNE were transformed into the *E*. *coli* strain C43 (DE3). pKAN6B-EcRraA was transformed into the *E*. *coli* strain DH5α. Each strain was grown in 2.0 L of LB liquid medium including appropriate antibiotics until the OD_600_ reached approximately 0.6, and 0.5 mM IPTG was added for inducing protein expression except for EcRraA that was induced by 0.2% L-arabinose. Five hours after induction at 30°C, cells were harvested and lysed with 50 ml lysis buffer (20 mM Tris-HCl pH 8.0, 300 mM NaCl, and 2 mM 2-mercaptoethanol). Then, cells were homogenized via sonication and target proteins were purified using a TALON^®^ metal affinity resin column (GE Healthcare, USA). 0.5 mM EDTA was added for preventing the impact of metal for the oligomerization of VvRraA1, VvRraA2, their C9D derivatives, and EcRraA, then they were further purified by HiLoad Superdex 200 26/600 (GE Healthcare, USA), pre-equilibrated with the lysis buffer containing 5% glycerol. The purified proteins were concentrated and stored frozen at -80°C until use.

For purification of pGEX-VvRNE, overexpression of GST-VvRNE was induced with 0.1mM IPTG at an OD_600_ of 0.6 and the cells expressing GST-VvRNE were incubated at 30°C for 3 hours. Collected cells were suspended in lysis buffer (20 mM Tris-HCl pH 8.0, 150 mM NaCl, and 2 mM 2-mercaptoethanol) and disrupted in a constant pressure (Constant Systems Ltd). After centrifugation of the lysate at 20,000 × g for 30 min, the supernatant was loaded onto a glutathione Sepharose 4B resin (GE healthcare) and the protein was eluted with 20 mM reduced GSH (glutathione) in the lysis buffer. The eluted protein was dialyzed against 20 mM Tris-HCl pH 8.0, 300 mM NaCl, and 2 mM 2-mercaptoethanol and then concentrated and stored frozen at -80°C until use.

### *In vitro* pull-down assay

The proteins of hexahistidine-tagged VvRraA1, VvRraA1-C9D, VvRraA2, VvRraA2-C9D (wild-type and mutants), and the GST-fused VvRne (612–816 region) were expressed and purified as described above. The GST-fused VvRne protein was bound to 10 μl of GSH resin and incubated with VvRraA1, VvRraA2, and their C9D derivatives (wild-type or mutants) at 4°C for 1 h. The resin was washed with 20 mM Tris-HCl (pH 8.0) buffer containing 300 mM NaCl and 2 mM 2-mercaptoethanol, and it was then eluted with the same buffer supplemented with 20 mM GSH. The eluted fractions were analyzed via sodium dodecyl sulfate polyacrylamide gel electrophoresis (SDS-PAGE), and the protein bands were stained with Coomassie blue.

### Oligomeric state determination

Size exclusion chromatography (Superdex 200 Increase 5/150 GL column, GE healthcare) equipped with multi-angle light scattering (MALS) instrument (WYATT, USA) was employed. The protein samples were applied to the Superdex 200 column in a buffer containing 20 mM Tris-HCl (pH 8.0), 300 mM NaCl, 5% glycerol, and 2 mM 2-mercaptoethanol. VvRraA1, VvRraA1-C9D, VvRraA2, VvRraA2-C9D, and *E*. *coli* RraA were diluted to 3 mg/ml in the corresponding buffer and then loaded onto the column. Data analyses were performed with ASTRA 6 software (WYATT, USA).

### *In vitro* cleavage of RNase E substrate

*In vitro* cleavage of BR10+hpT has been described previously [6].

## Results

### Substitution of Cys9 residue of VvRraA1 and VvRraA2 with Asp affects oligomerization status of the proteins

**Fig 2:**
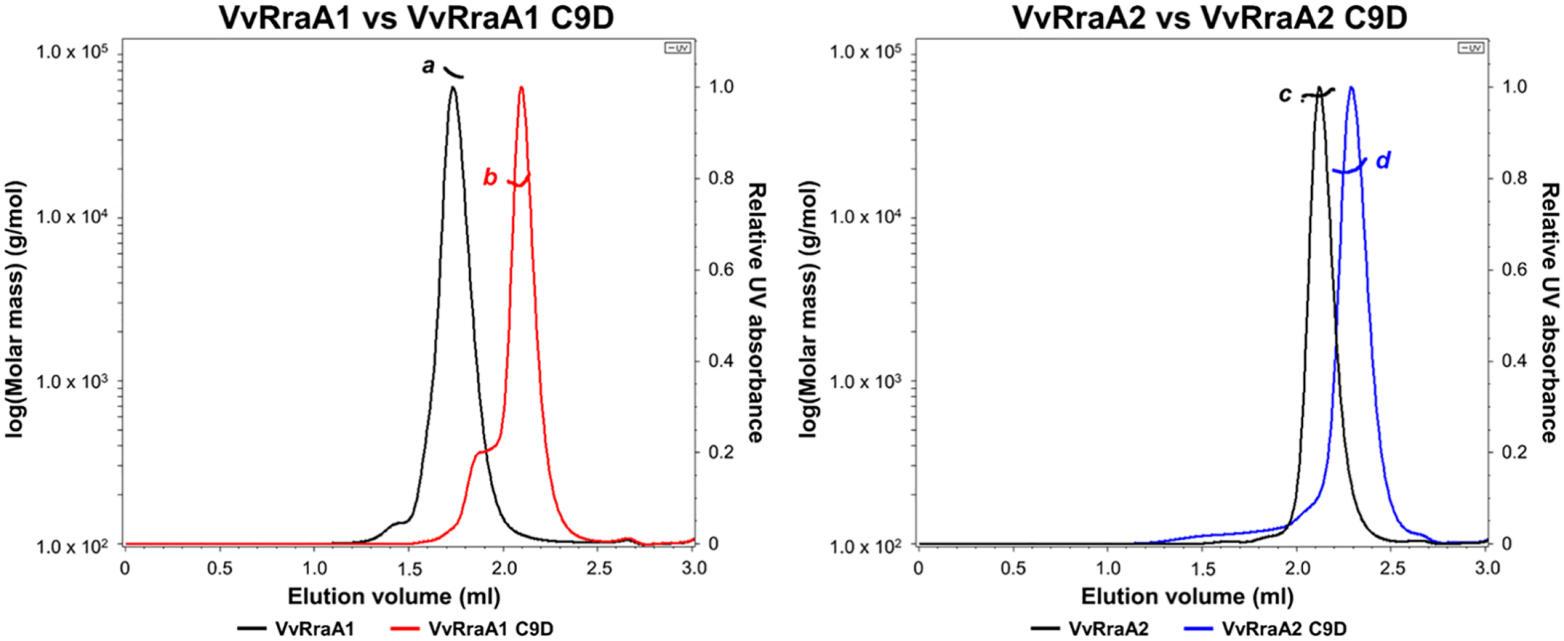
Oligomerization state of VvRraA proteins. Size exclusion chromatography with MALS of VvRraA proteins and their C9D mutants. The molecular sizes of the peaks were estimated by using MALS, as indicated. Based on these results, *a*, corresponds to 75.62 kDa (~hexamer); *b*, 16.3 kDa (monomer); *c*, 56.64 kDa (trimer); *d*, 19.68 kDa (monomer). Although the peak ‘*a*’ for the wild-type VvRraA1 is in between hexameric and trimeric sizes (~4.7 times higher than the monomeric mutant), it is most likely that VvRraA1 is a hexameric form since all the native RraA proteins are in the forms of a trimer or a hexamer.

To investigate functional role of oligomerization of VvRraA proteins, we searched for amino acid residues whose substitutions may affect oligomerization of VvRraA proteins. We found that two cysteines, Cys9 and Cys41, are conserved in both VvRraA proteins as well as in RraA homologs found in Gram-negative bacteria (Fig 1A). We began by investigating the possible structural changes of VvRraA proteins caused by mutation of the Cys9 residue because structural modeling study based on the known structures of RraA homologs suggested that the Cys9 residue is located at the interface between the protomers (Fig 1B). To investigate the role of Cys9, the residue was substituted with aspartate (Asp) in VvRraA1 and VvRraA2 (VvRraA1-C9D and VvRraA2-C9D, respectively). By using the elution profile of size exclusion chromatography with multi-angle light scattering (SEC-MALS), it was shown that the C9D mutant proteins were completely different from wild-type VvRraA proteins in terms of oligomerization (Fig 2). In the case of VvRraA1, wild-type VvRraA1 existed in a hexameric state in solution, whereas hexameric oligomers of VvRraA1-C9D were disrupted as to exist in monomer. Wild-type VvRraA2 also existed as a trimer in solution but VvRraA2-C9D was revealed to undergo a monomer conversion. These results imply that an amino acid substitution of the most conserved cysteine residue to aspartic acid (C9D) interferes with the oligomerization of VvRraA proteins.

### Effects of VvRraA1, VvRraA2, and C9D mutant co-expression on growth of *E*. *coli* cells overproducing VvRNase E

**Fig 3:**
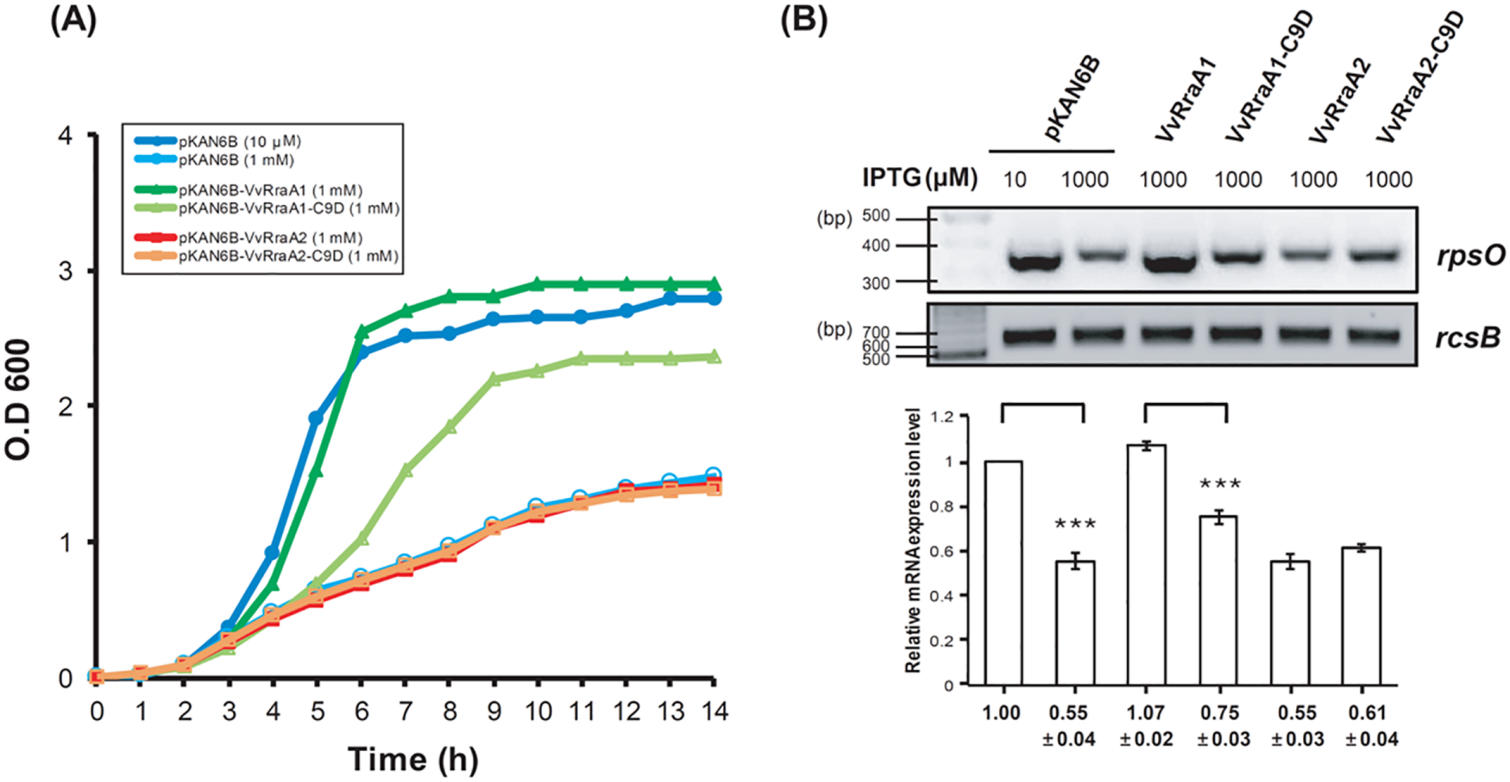
Effects of the C9D mutation on the activity of VvRraA proteins *in vivo*. (A) Effects of co-expression of VvRraA proteins on growth in *E*. *coli* cells overproducing VvRNase E. The cultures of DK001 cells containing pKAN6B, pKAN6B-VvRraA1, pKAN6B-VvRraA1-C9D, pKAN6B-VvRraA2, or pKAN6B-VvRraA2-C9D were grown in LB-10 μM IPTG medium and 0.2% arabinose and no additional IPTG (DK001+ pKAN6B+ 10 μM IPTG) or 1 mM IPTG (DK001+ pKAN6B+1 mM, DK001+ pKAN6B-VvRraA1, DK001+ pKAN6B-VvRraA1-C9D, DK001+ pKAN6B-VvRraA2, and DK001+ pKAN6B-VvRraA2-C9D) were added to the cultures at OD_600_ = 0.1~0.2. Then, their growth was monitored by analyzing cell density (absorbance at 600 nm) at specific time intervals. (B) Effects of co-expression of VvRraA proteins on the steady-state level of *rpsO* mRNA in DK001. Total RNA was isolated from DK001 cells grown to an OD_600_ of 1.5 in the same manner described in Fig 3A, and reverse transcription-polymerase chain reaction (RT-PCR) was performed. The relative abundance of each mRNA is shown at the bottom of the gels. Student’s t-test was used for comparisons with control using SAS version 9.2 (SAS Institute, Cary, NC, USA). The data were repeated three times and presented as mean ± SEM. ***p < 0.001.

To test whether structural changes in VvRraA1 protein affect its known inhibitory effect on ribonucleolytic activity of VvRNase E (VvRne) [6], and whether VvRraA2 and VvRraA2-C9D can modulate the activity of VvRne, we investigated *in vivo* activity of these VvRraA proteins. We first assessed the growth rate of DK001 cells co-expressing VvRraA1, VvRraA2, or C9D mutants. DK001 contains a deletion in the chromosomal *rne* gene that is complemented by plasmid-borne VvRne (pLAC-RNEV2), which expresses a full-length VvRne with a hexahistidine tag at the C-terminus under the control of an IPTG-inducible *lacUV5* promoter [6]. To co-express the VvRraA proteins and their C9D mutants, a compatible Km^r^ plasmid that expresses VvRraA1, VvRraA1-C9D, VvRraA2, and VvRraA2-C9D with hexahistidine tags at their C-termini under the control of an arabinose-inducible promoter (pKAN6B-VvRraA1, pKAN6B-VvRraA2, pKAN6B-VvRraA1-C9D, or pKAN6B-VvRraA2-C9D, respectively) was introduced into DK001 cells. The growth of DK001 cells was inhibited by overexpression of VvRne in the presence of 1 mM IPTG compared to cells in the presence of 10 μM IPTG, as has been reported previously [6]. Co-expression of VvRraA1 rescued DK001 cells from growth retardation caused by overexpression of VvRne in the presence of 1 mM IPTG, showing growth rates comparable with those of DK001 cells optimally expressing VvRNase E in the presence of 10 μM IPTG (Fig 3A). Co-expression of VvRraA1-C9D was not able to effectively rescue DK001 cells overexpressing VvRne from growth retardation as co-expression of VvRraA1. On the contrary, co-expression of VvRraA2 or VvRraA2-C9D did not affect growth rates of DK001 overexpressing VvRne. These results indicate that (i) VvRraA1 can effectively modulate the activity of over-produced VvRNase E, rendering VvRne-overproducing cells to normally grow, and this VvRraA1 activity is largely abolished by the C9D mutation. (ii) Co-expression of VvRraA2 and VvRraA2-C9D does not inhibit the enzymatic activity of VvRne in VvRne-overproducing cells.

### Effects of VvRraA1, VvRraA2, and their C9D mutant co-expression on the ribonucleolytic activity of VvRNase E *in vivo*

To test whether VvRraA1-C9D, VvRraA2, and VvRraA2-C9D are able to regulate the ribonucleolytic activity of VvRne *in vivo*, *rpsO* mRNA, one of the known substrates of RNase E in *E*. *coli*, was analyzed for their steady-state levels by semi-quantitative RT-PCR (Fig 3B). The results showed that co-expression of VvRraA1 effectively inhibited VvRne action on *rpsO* mRNA in DK001 cells overproducing VvRne, causing the increased level of the RNA transcripts *in vivo*, while co-expression of VvRraA1-C9D less effectively inhibited VvRne action in these cells. In contrast, no significant changes in the level of *rpsO* mRNA were observed when either VvRraA2 or VvRraA2-C9D was co-expressed in VvRne-overproducing DK001 cells. These results indicated that the inhibitory activity of VvRraA1-C9D on VvRNase E action was more decreased than that of VvRraA1, whereas VvRraA2 or VvRraA2-C9D could not modulate the ribonucleolytic activity of VvRNase E on *rpsO* mRNA *in vivo*.

### Physical interactions of VvRraA proteins with VvRNase E

**Fig 4:**
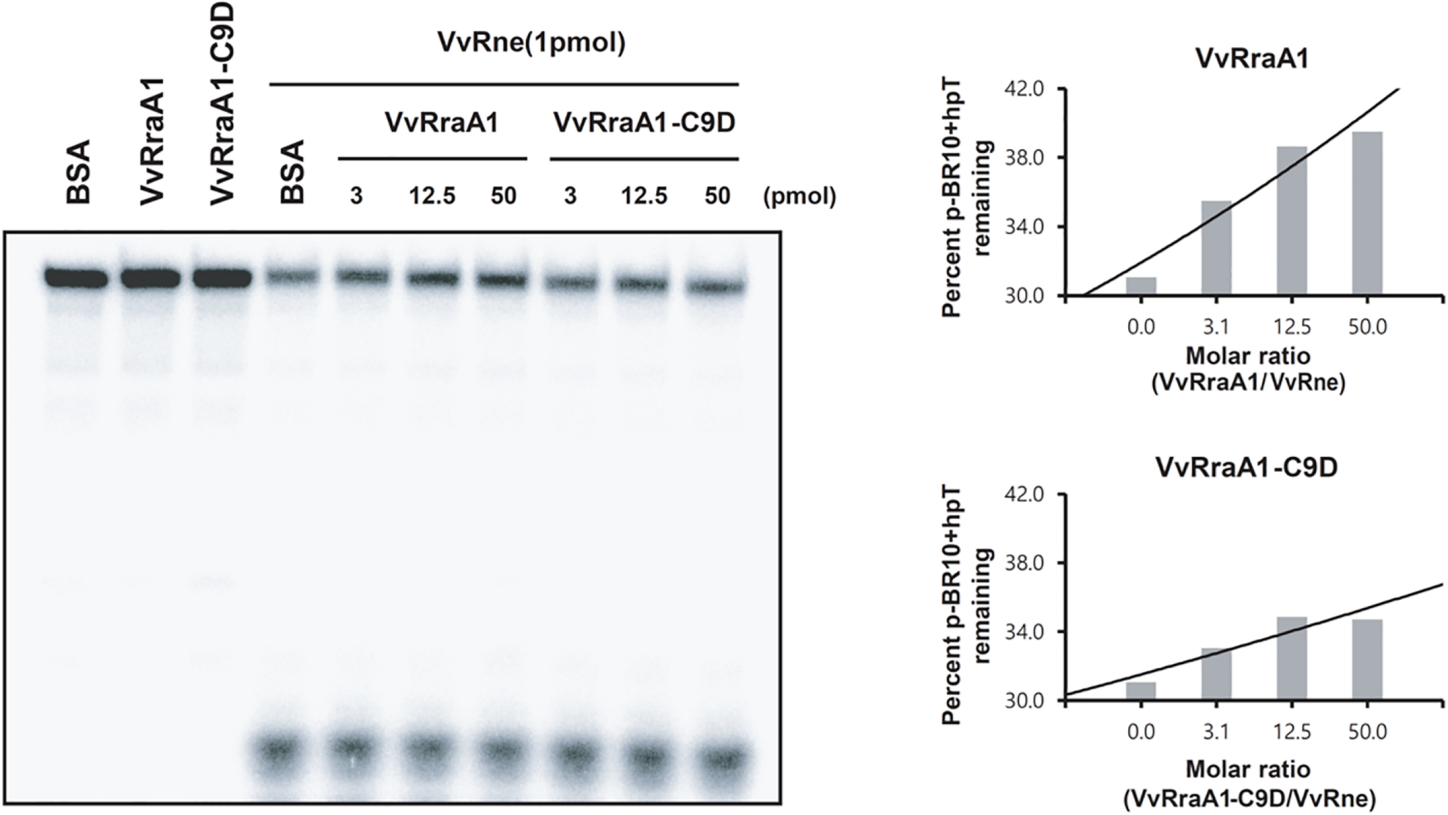
Interactions of VvRNase E with VvRraA proteins. Hexahistidine-tagged VvRraA1, VvRraA1-C9D, VvRraA2, VvRraA2-C9D, and the GST-fused VvRne (612–816 residues) were expressed and purified as described in the Methods section. The GST-fused VvRne protein was bound to GSH resin and incubated with VvRraA proteins and their C9D mutant proteins. Then, the proteins were eluted and the fractions were analyzed using SDS-PAGE. The protein bands were stained with Coomassie blue. Only VvRraA1 could tightly bind to VvRne.

To explain the reason for the observed differential inhibitory activity of VvRraA1, VvRraA1-C9D, VvRraA2, and VvRraA2-C9D on VvRNase E, the *in vitro* physical interactions between VvRraA proteins and VvRne were investigated via pull-down assays by using GST tags. Because it is already known that the VvRraA1 protein interacts with the C-terminal region of VvRNase E, the C-terminal region (612–816 residues) of VvRNase E protein fused with a GST tag was used for these experiments. This short VvRNase E protein and hexahistidine-tagged VvRraA1, VvRraA1-C9D, VvRraA2, and VvRraA2-C9D were expressed and purified as described in the Methods section. We found that only VvRraA1 was tightly bound to VvRNase E, implying that the hexamerization of VvRraA1 is important for interaction of the protein with VvRNase E, and subsequent inhibitory function on VvRNase E activity (Fig 4).

### Inhibition of the ribonucleolytic activity of VvRNase E by VvRraA1 and VvRraA1-C9D *in vitro*

**Fig 5:**
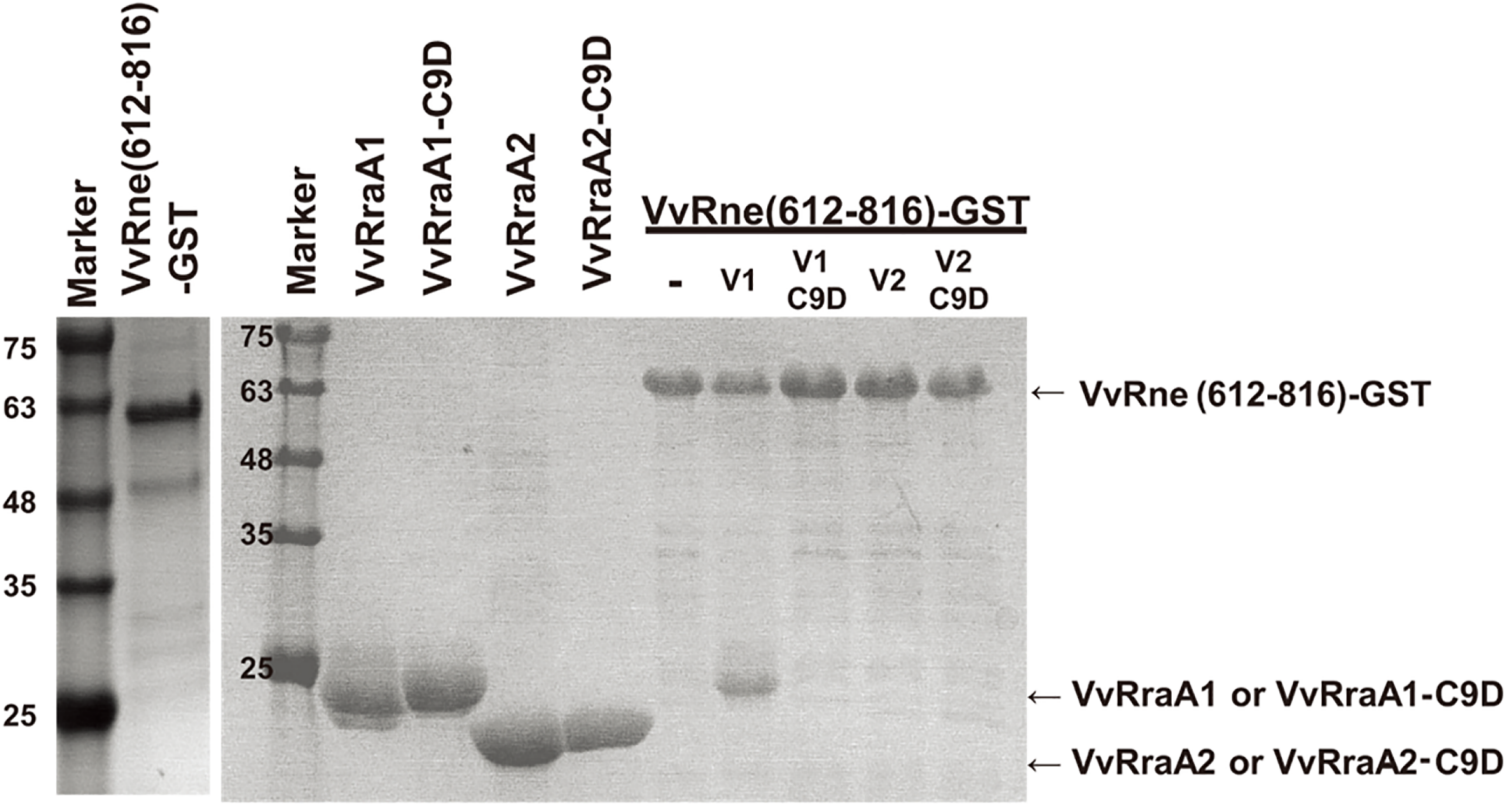
Inhibition of VvRraA1 and VvRraA1-C9D on the cleavage of p-BR10+hpT by VvRNase E *in vitro*. 0.5 pmol of 5’-end-labeled p-BR10+hpT RNA was incubated with 1 pmol of VvRne with varying concentrations of VvRraA1 and VvRraA1-C9D, 50 pmol of VvRraA1, or 50 pmol of BSA in 20 μl of 1 × cleavage buffer at 37°C for 2 h for VvRne, VvRraA1 only, or BSA only controls. Samples were mixed with an equal volume of loading buffer, and then denatured at 65°C for 5 min and loaded onto a 12% polyacrylamide gel containing 8 M urea. The percentage of uncleaved p-BR10+hpT in the gel was quantitated using a phosphorimager and OptiQuant software.

Because VvRraA1 and VvRraA1-C9D showed differential inhibitory effects on ribonucleolytic activity of VvRne while VvRraA2 and VvRraA2-C9D did not, we further studied the inhibitory effects of VvRraA1 and VvRraA1-C9D on the ribonucleolytic activity of VvRNase E *in vitro*. In this experiment, we also wished to examine whether the decreased inhibitory effect of VvRraA1-C9D on the VvRNase E activity *in vivo* is a direct consequence of its inefficient inhibition of the ribonucleolytic activity of VvRNase E on RNA substrates. VvRne, VvRraA1, and VvRraA1-C9D were affinity-purified for *in vitro* cleavage assays. A 5′-^32^P-end-labeled BR10+hpT (p-BR10+hpT) was used as a VvRNase E substrate. This RNA substrate is a synthetic oligonucleotide containing the RNA I cleavage site of RNase E and surrounding nucleotides located at its 5′-end and 3′-end, corresponding to the distal hairpin loop and transcription termination regions in each [6]. As shown in Fig 5, VvRraA1-C9D also leads to a direct inhibition of the ribonucleolytic activity of VvRne in a dose-dependent manner, but a lower degree of inhibition was shown compared with VvRraA1. These results show that the decreased inhibitory effect of VvRraA1-C9D on the VvRne action *in vivo* stems from its inefficient inhibition of the ribonucleolytic activity of VvRne, and its action does not require other factor(s).

## Discussion

Recently, the significance of RNase E in the regulation of gene expression via RNA decay and processing has been widely studied in *E*. *coli*. It has been revealed that the marine pathogenic bacterium *V*. *vulnificus* has functional orthologs of RNase E and its inhibitor RraA [6, 18, 19]. In this study, we used the mutant proteins of VvRraA1 and VvRraA2 that contain the C9D substitution to investigate the structural elements required for the inhibition of VvRNase E activity. We found that VvRraA1, which existed in the hexameric form in solution, efficiently inhibited the enzymatic activity of VvRNase E, whereas the inhibitory effect of VvRraA1-C9D, which existed as a monomer, on VvRNase E action was reduced *in vitro* and *in vivo*. VvRraA2 and VvRraA2-C9D, which existed as a trimer and monomer in solution, respectively, did not inhibit the enzymatic activity of VvRNase E. In the *in vitro* GST pull-down assay, only VvRraA1 directly bound to VvRNase E, implying that the inhibitory effect was affected by the binding efficiency of VvRraA proteins to VvRNase E. These findings reveal how VvRraA proteins actively regulate VvRNase E activity on its substrate RNA in *V*. *vulnificus*, suggesting that the hexamerization of VvRraA proteins is required for their inhibition activity on VvRNase E action. Further studies of these RraA homologs in *V*. *vulnificus* are required to identify their physiological role in this marine pathogenic bacterium.

**Fig 6:**
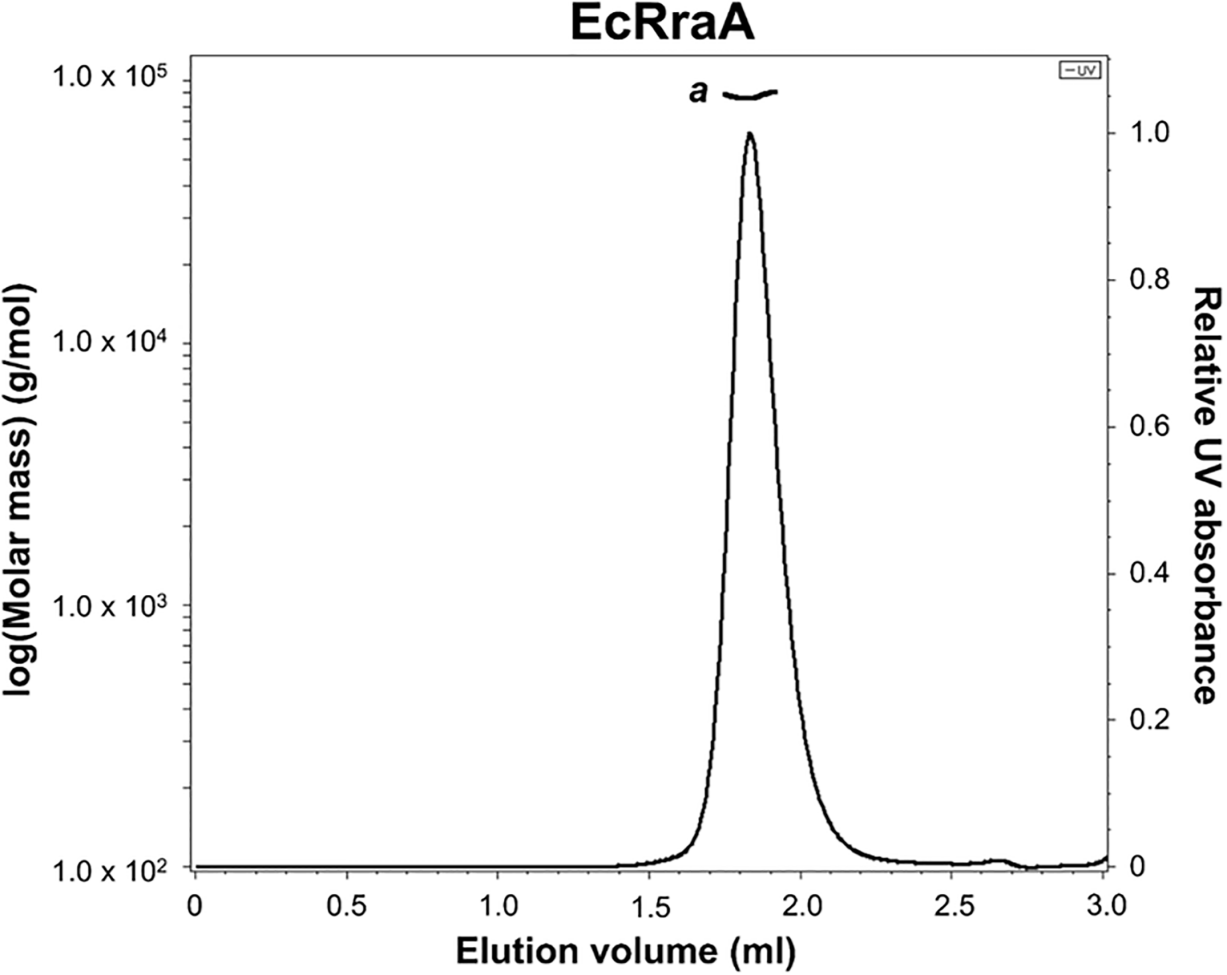
Oligomerization state of EcRraA protein. Size exclusion chromatography with MALS of EcRraA was performed as described in the legend to Fig 2. *a*, 87.05 kDa (~hexamer).

Considering that reported crystal structures of five RraA homologs share trimer assembly, except for RraA proteins from *P*. *aeruginosa* and *S*. *coelicolor*, it is difficult to generalize hexamerization of RraA homologs is required for their inhibitory activity on RNase E-like proteins. However, it is worthwhile to note that the inhibitory activity of these RraA homologs on RNase E-like proteins has not been evaluated except for EcRraA and ScRraA2 [2]. In addition, we found that EcRraA, whose X-ray structure shows a ring-like trimer [10], existed in a hexamer state in solution when the elution profile of SEC-MALS was analyzed (Fig 6). It has been reported that higher concentration of ammonium acetate promoted formation of EcRraA hexamer [11]. However, we observed that EcRraA and VvRraA1 that retain full inhibition activity to their RNase E-like proteins form the hexamer in the solution without ammonium acetate in the SEC-MALS data (Figs 2 and 6). Thus, all three RraA homologs (EcRraA, ScRraA2, and VvRraA1) that have been evaluated and shown for their effective inhibitory activity on RNase E-like proteins display hexameric assembly [2, 6, 21]. In supporting this notion, the RraA hexameric assembly, which is mediated by a dimer of trimers, is found in all crystal structures of RraA homologs available in PDB database even though the hexamer appears to be present in high salt conditions *in vitro*. These RraA homologs require scaffold domains of RNase E-like enzymes for high-affinity binding and inhibitory action on the ribonucleolytic activity. Based on these data, we postulate that hexameric forms of RraA, which may not be as dominant as trimer forms under physiological conditions, more efficiently facilitate efficient binding to RNase E-like proteins and, in turn, inhibitory activity on the action of RNase E-like enzymes. In addition, structural studies on ScRraA1, another *S*. *coelicolor* RraA homolog, which uniquely interacts with the catalytic domain of RNase ES and inhibits its ribonucleolytic activity [22], will further unveil structural features of the interaction between RraA homolog and RNase-E like enzymes.
