# Correction: Functional implications of hexameric assembly of RraA proteins from *Vibrio vulnificus*

**DOI:** 10.1371/journal.pone.0191775

**Published:** 2018-01-19

**Authors:** Saemee Song, Seokho Hong, Jinyang Jang, Ji-Hyun Yeom, Nohra Park, Jaejin Lee, Yeri Lim, Jun-Yeong Jeon, Hyung-Kyoon Choi, Minho Lee, Nam-Chul Ha, Kangseok Lee

The images for Figs 4 and 5 are incorrectly switched. The image that appears as Fig 4 should be Fig 5, and the image that appears as Fig 5 should be Fig 4. The figure captions appear in the correct order. Please see the corrected Fig 4 and Fig 5 below.

**Fig 4:**
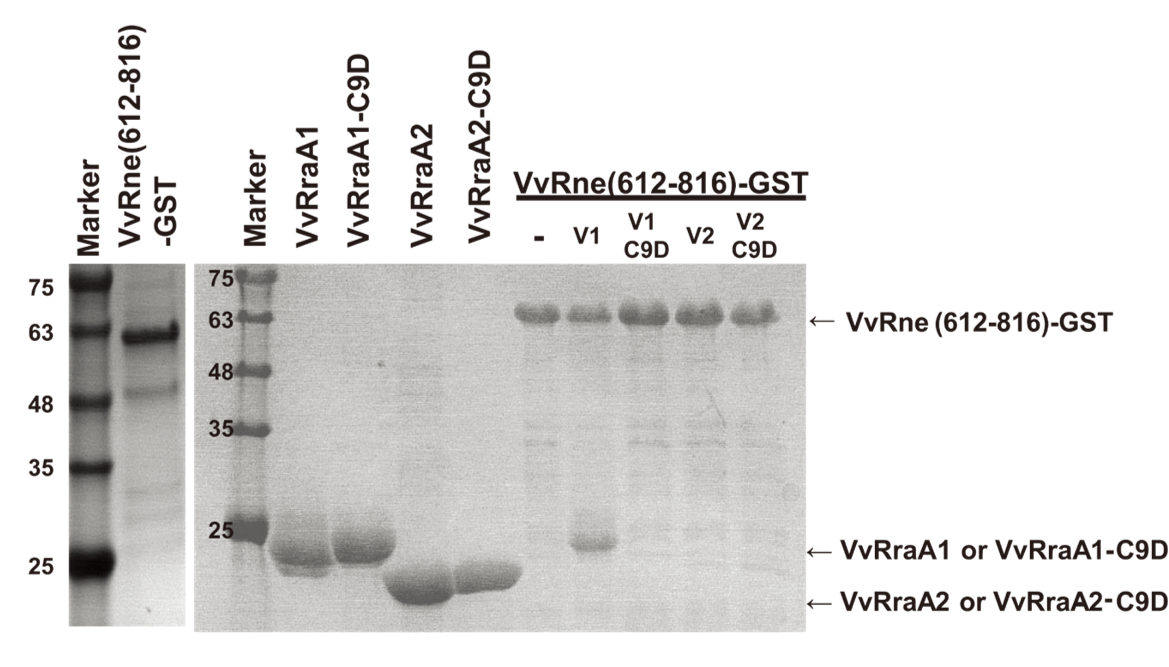
Interactions of VvRNase E with VvRraA proteins. Hexahistidine-tagged VvRraA1, VvRraA1-C9D, VvRraA2, VvRraA2-C9D, and the GST-fused VvRne (612–816 residues) were expressed and purified as described in the Methods section. The GST-fused VvRne protein was bound to GSH resin and incubated with VvRraA proteins and their C9D mutant proteins. Then, the proteins were eluted and the fractions were analyzed using SDS-PAGE. The protein bands were stained with Coomassie blue. Only VvRraA1 could tightly bind to VvRne.

**Fig 5:**
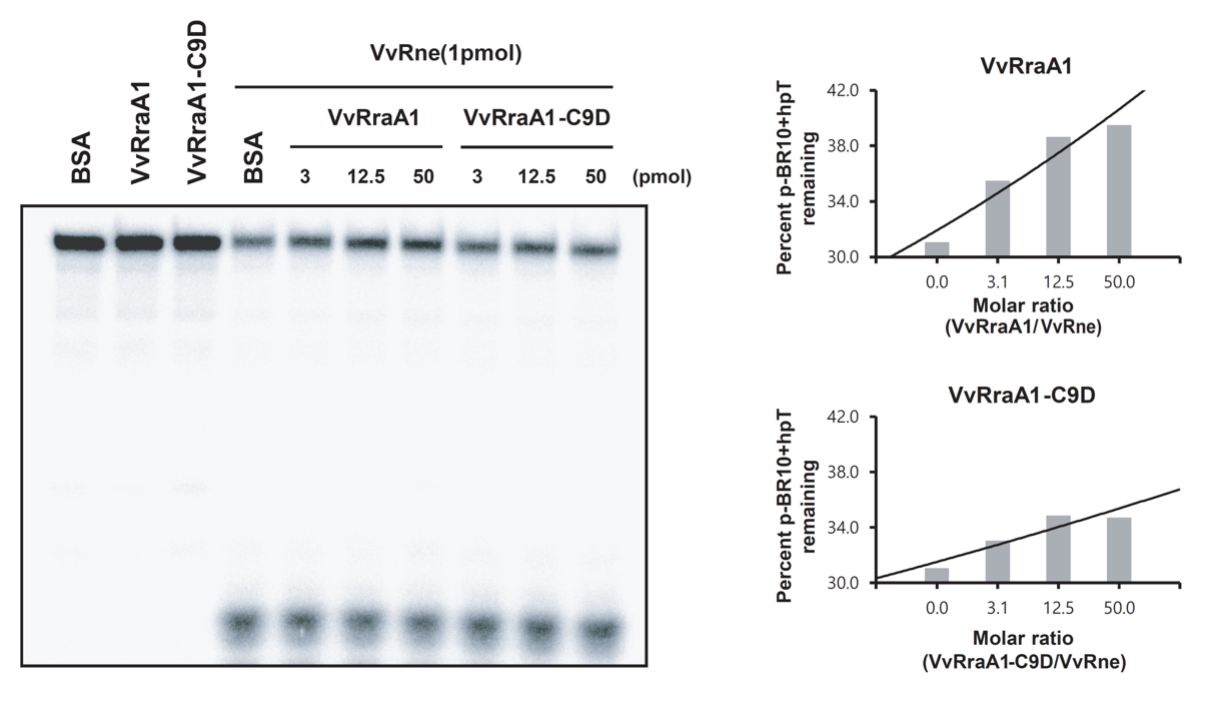
Inhibition of VvRraA1 and VvRraA1-C9D on the cleavage of p-BR10+hpT by VvRNase E *in vitro*. 0.5 pmol of 5’-end-labeled p-BR10+hpT RNA was incubated with 1 pmol of VvRne with varying concentrations of VvRraA1 and VvRraA1-C9D, 50 pmol of VvRraA1, or 50 pmol of BSA in 20 μl of 1 × cleavage buffer at 37°C for 2 h for VvRne, VvRraA1 only, or BSA only controls. Samples were mixed with an equal volume of loading buffer, and then denatured at 65°C for 5 min and loaded onto a 12% polyacrylamide gel containing 8 M urea. The percentage of uncleaved p-BR10+hpT in the gel was quantitated using a phosphorimager and OptiQuant software.
